# Dissolution Rate Enhancement, Design and Development of Buccal Drug Delivery of Darifenacin Hydroxypropyl *β*-Cyclodextrin Inclusion Complexes

**DOI:** 10.1155/2013/983702

**Published:** 2012-10-22

**Authors:** Swati C. Jagdale, Prachyasuman Mohanty, Aniruddha R. Chabukswar, Bhanudas S. Kuchekar

**Affiliations:** Department of Pharmaceutics, MAEER's Maharashtra Institute of Pharmacy, S. Number 124, MIT Campus, Kothrud, Maharashtra, Pune 411 038, India

## Abstract

Darifenacin is a urinary antispasmodic. The oral absorption of darifenacin is poor due to its low solubility and poor bioavailability (15–19%). Darifenacin was complexed with hydroxylropyl beta-cyclodextrin (Hp*β*-CD). The best results were obtained with the coevaporation that interacts in a 1 : 1 drug : cyclodextrin molar ratio. The solid inclusion complexes were found to be amorphous in the characterization. The dissolution rate of darifenacin from the Hp*β*-CD solid inclusion complex was increased compared to the powdered drug. The controlled release buccoadhesive patches for the delivery of darifenacin were prepared using HPMC K100M CR and HPMC K15. The coevaporation complex of the drug was used in the formulation due to its increased saturation solubility and increased ease of dissolution. The patches were evaluated for their surface pH, folding endurance, swelling, mucoadhesive properties, *in vitro* residence time, vapour transmission test, and *in vitro* and *ex vivo* release studies. Formulations Hb2 (2%) and Pb4 (4%) were found to be optimized. These two formulations can be used for buccal delivery of darifenacin which avoids first pass effect and leads to increased bioavailability of darifenacin.

## 1. Introduction

Cyclodextrin is capable of forming inclusion complexes with many drugs by taking up a whole drug molecule, or a part of it, into the cavity of the cyclodextrin molecule. Drug cyclodextrins complexes can improve the clinical usage of drugs by increasing their aqueous solubility, dissolution rate, and pharmaceutical availability [1]. Hp*β*-CD can be used to solubilise poorly water-soluble drugs by complexation and then delivery via the buccal or sublingual mucosa may be advantageous for increasing drug absorption. The buccal route has high acceptance due to avoidance of first pass metabolism and possibility of being accessible for controlled drug release. Various bioadhesive mucosal dosage forms have been developed which include adhesive tablets, gels, ointments, and more recently patches. Buccal patches are preferred over adhesive tablets in terms of flexibility and patients comfort.Nowadays bioadhesive polymers receive considerable attention as platforms for buccal controlled delivery due to their ability to localize the dosage form in specific regions to enhance drug bioavailability [2, 3].

Buccal mucosa is a potential site for the delivery of drugs to the systemic circulation. A drug administered through the buccal mucosa enters directly the systemic circulation, thereby minimizing the first-pass hepatic metabolism and adverse gastrointestinal effect, for example, diltiazem hydrochloride, and testosterone [1]. Buccal permeation can be improved by using various classes of transmucosal and transdermal penetration enhancers such as bile salts, surfactants, fatty acids and derivatives, chelators, and cyclodextrin [4].

 In recent years, mucoadhesive patches have attracted considerable attention as sustained drug release devices. Various types of polymers can be used in the buccal patches and the hydration of these polymers results in the formation of an outer gel layer that controls drugs release. HPMC, the non-ionic cellulose ether, is commonly used in the formulation of mucoadhesive patches. Drug carrier systems such as drug-cyclodextrin complexes incorporated in hydrophilic patches could provide controlled and complete *in vitro *drug release.

Darifenacin is used for the treatment of overactive bladder, with symptoms of urge urinary incontinence, urgency, and frequency.Urge urinary incontinence is involuntary urination immediately following an urge to urinate. Urgency is the feeling of needing to urinate, and frequency is an increase in how often a person feels the urge to urinate. Darifenacin is given once daily (7.5 mg dose) [5]. Darifenacin shows 98% (primarily alpha_1_-acid glycoprotein) protein binding. Half life of darifenacin is 13–19 hours and bioavailability of darifenacin is very low (15–19%) due to low solubility and high first pass metabolism in oral route of administration. Therefore, the aim of the present work was to study the complexation of darifenacin with hydroxyl propyl beta-cyclodextrin to enhance the solubility of drug,and to develop buccal controlled-release formulation to avoid first pass effect and increase bioavailability.

## 2. Material and Method

### 2.1. Material

Darifenacin was kindly gifted by Microlab Ltd. Bangalore. Hydroxypropyl beta-cyclodextrin (Hp-*β*CD) was gifted by Gangwal chemicals, Mumbai. HPMC K100 M CR and HPMC K15 were gifts from Colorcon India, Mumbai. All other materials and solvents used were of analytical reagent grade.

### 2.2. Drug Characterization

Melting point of darifenacin was determined by capillary method.Solubility of darifenacin is checked in various solvents. Infrared spectrum was recorded using FTIR spectrophotometer (Varian 640 IR spectrophotometer (Varian, Australia)). The UV spectrum (Varian Cary 100) was recorded in the range 200–400 nm preparing solution of darifenacin 10–50 *μ*g/mL in distilled water, pH 6.8 buffer, and 0.1 N HCl solvent. The wavelength maximum absorption (*λ*_max_) was found from the scan and then further preparation of calibration (standard) curve was carried out at the detected wavelength of maximum absorption (*λ*_max_). 

### 2.3. Part I

#### 2.3.1. Phase Solubility Studies

Phase solubility studies were performed by the method previously reported by Higuchi and Connors [6]. Briefly, excess amounts of darifenacin were added to 20 mL of aqueous solutions containing various concentrations of Hp*β*CD (2.5 × 10^−3^ to 15 × 10^−3 ^M). The suspensions were vigorously shaken at 25 ± 1°C for 3 days. After equilibrium was attained, the samples were filtered through a 0.45 m Millipore membrane filter and suitably diluted with distilled water. Darifenacin concentration was determined spectrophotometrically, *λ*_max_ = 285 nm. The apparent 1 : 1 stability constant, *K*_*s*_, was calculated from the phase solubility diagrams using the equation:

(1) $$\begin{array}{c} K_{s}=\frac{\text{slope}}{S_{0}\left(1-\text{slope}\right)}, \end{array}$$

where *S*_0_ is the solubility of darifenacin in the absence of Hp*β*CD (intercept).

#### 2.3.2. Stability Study of Drug in Different Solvents and Calibration Curve

Darifenacin is incorporated in 0.1 N HCl,pH 6.8 buffer, and distilled water sonicated for 10 minutes and analysed after 48 hours in UV spectrophotometer and analysed for shift in absorption maxima or decrease in absorbance. The calibration curves were prepared using the above solvents and preparing solution of darifenacin 10–50 *μ*g/mL range and the UV spectrum was recorded in the range 200–400 nm.

#### 2.3.3. Preparation of Solid Complexes

Complexes were prepared in 1 : 1 darifenacin: Hp*β*-CD molar ratio based on the results of the solubility studies.

*Physical Mixture (PM). *The physical mixtures of drug and Hp*β*-CD (1 : 1 molar ratio) were obtained by mixing pulverized powder (no. 100) together in pestle and mortar at room temperature [7]. 

*Kneading Method (Kn).* Drug & Hp*β*-CD triturated in a mortar with a small volume of water-methanol solution. The thick slurry was kneaded for 45 min and then dried at 40°C. Dried mass was pulverized and sieved through (no. 100) [7]. 

*Co-Evaporation Method (COE). *The aq. solution of Hp*β*-CD was added to an alcoholic solution of drug. The resulting mixture was stirred for 1 hr & evaporated at a temperature of 45°C until dry. The dried mass was pulverized & sieved through (no. 100) [7]. 

*Co-Grounding (COG). *Drug was triturated with minimum quantity of methanol in a glass mortar until it dissolved. Then Hp*β*-CD was added and suspension was triturated rapidly at room temperature until solvent is evaporated [8]. 

*Freeze-Drying Method (FD). *Physical mixtures of drug & Hp*β*-CD in a molar ratio of 1 : 1 were added to 500 mL double distilled water and stirred for 5 days. The suspension was freeze-dried (ilshin freeze dryer), and the obtained freeze-dried complex was pulverized and sieved through (<38 *μ*m) [9]. 

*Melting Method (MELT).* Melting method was used for the preparation of drug-Hp*β*-CD complex. The drug-Hp*β*-CD ratio (1 : 1 molar) was accurately weighed, mixed in crucible, and the mixture was kept in ampoule for melting. The mixture was cooled slowly at room temperature. The product was placed in desiccators. The solidified product was transferred to a clean mortar, triturated, and passed through sieve no.16 and 20 [10]. 

*Spray Drying Method (SPD). *A mixture of drug, Hp*β*-CD was dissolved in 250 mL of water. The resultant solution was spray dried using a spray dryer. The spray drying was done at the following sets of conditions; air flow rate at 400 Nl/h, spray nozzle with a diameter 0.7 mm under the atomization pressure of 2 kg/cm^2^ with a feed rate of 4 mL/min. The inlet temperature was kept at 120°C and out let temperature 90°C ± 2°C. The vacuum in the system was 60 mmWc and aspiration rate was 40 m Bar. The product thus obtained was collected, packed, doubly wrapped in an aluminium foil and stored in a desiccator till further use [11].

#### 2.3.4. Evaluation of Complexes

*Percentage Yield Study.* The prepared complexes were weighed and the yield was calculated for each preparation using the following formula

(2) $$\begin{array}{c} \%\;\text{Yield}=\left(\frac{a}{b}\right)\times 100, \end{array}$$

where, “*a*” is the practical weight of complex obtained and “*b*” is the theoretical weight of complex prepared [12].

*Determination of Drug Content of Complex. *Drug: Hp*β*-CD complex equivalent to 10 mg of drug was stirred with 100 mL of methanol for 60 min; the solution was filtered and treated as stock solution containing 100 *μ*g/mL drug. From this stock solution the concentration of 10 *μ*g/mL was prepared and the drug content was determined spectrophotometrically at 285 nm [13]. 

*Saturation Solubility Studies. *Excesss amount of drug, PM, and inclusion complexes were added to the 250 mL conical flasks containing 25 mL of double distilled water. The sealed flasks were shaken for 48 h at room temperature followed by equilibrium for three days. The aliquots were withdrawn through whatman filter paper and determined by UV spectrophotometry [14]. 

*Differential Scanning Calorimetry (DSC). *Differential scanning calorimetry (DSC) has been one of the most widely used calorimetric techniques to study the solid state interaction of drug with Hp*β*-CD [14]. Samples of the solid complexes, pure drug, and *β*-CD were taken in flat bottomed aluminium pans and heated over a temperature range of 25 to 300°C at a constant rate of 10°/min with purging of nitrogen (50 mL/min) using alumina as a reference standard in a differential scanning calorimeter (DSC-7, Perkin Elmer).

*X-Ray Diffractometry*. Powder X-ray diffraction technique has been extensively utilized along with DSC to study the interaction between drug and Hp*β*-CD [15]. The diffraction studies were carried out in a powder X-ray diffractometer (STOE*­*STADI-P). The samples were rotated during data collection to reduce orientation effects. PXRD patterns of solid complex, pure drug, and Hp*β*-CD were recorded between 2*θ* = 5 to 50° at 40 kV and 30 mA. 

*FT-IR Spectroscopic Studies.* FT-IR has been employed as a useful tool to identify the drug excipient interaction [14]. Samples were analyzed by potassium bromide pellet method in an IR spectrophotometer (Varian, Australia) in the region between 4000 to 400 cm^−1^. Complex formation was evaluated by comparing the IR spectra of the solid complex and of the drug. 

*Dissolution Study of Complexes.* Dissolution study of the complexes of darifenacin Hp*β*-CD was done in pH 6.8 buffer using USP type II dissolution apparatus model TDT-08D [15]. Temperature was maintained at 37 ± 2°C. Dose-equivalent amount of complexes were taken and enclosed in basket. These bags were tied with paddles and dissolution was studied at 60 rpm for 2 h. 

*Stability Studies. *The selected formulations were packed in amber-colored bottles, which were tightly plugged with cotton and capped with aluminium. They were then stored at 25°C/60% RH, 30°C/65% RH, and 40°C/75% RH for 3 months and evaluated for their physical changes such as colour and texture, drug-cyclodextrin interaction, drug content, and dissolution study.

### 2.4. Part II

#### 2.4.1. Formulation

Coevaporation complex was optimised for the patch formulation due to very much increase in saturation solubility and percentage increase in solubility. Co-evaporation complex also shows higher increment in dissolution property of drug. Though spray dried and freeze dried products show more saturation solubility and dissolution rate than co-evaporation complexes they were not preferred as they were expensive and sophisticated process which may increase the formulation costs.

#### 2.4.2. Bioadhesive Patch Preparation

The patches were prepared by solvent casting method [16] using film forming polymer. The variables used while formulating the patches of HPMC K100CR/HPMC K15 and plasticizer (propylene glycol) concentrations (Tables 1 and 2). The concentration of HPMC K100CR/HPMC K15 was varied from 1 to 5%. The concentration of plasticizer was finalized differently depending on the concentration of polymer and from the plasticity of the films. It was varied from 20 to 30% for the patch. The samples were packaged in aluminium foil and stored in a glass container maintained at room temperature and 58% RH. For the medicated patches, calculated amount of drug-Hp*β*-CD complex (equivalent to 7.5 mg darifenacin) was incorporated in before addition of plasticizer and casting was performed in the same way as mentioned above. *In vitro *release studies of all the trial batches were done and Hb2 (2%) of HPMC K100CR patches and Pb4 (4%) of HPMC K15 patches were optimised due to their desired release profile.

#### 2.4.3. Evaluation of Polymer Films

Polymer films of 1 cm × 1 cm dimension were evaluated for the following tests.

*Patch Thickness.* Assessment of thickness was done on 5 patches from every batch by using micrometer screw gauge.

*Surface pH*. Agar plate was prepared by dissolving 2% (w/v) agar in warmed isotonic phosphate buffer of pH 7.4 under stirring and then pouring the solution in a petri dish and cooling till gelling at room temperature [16]. Buccal patches were left to swell for 2 hr on the surface of these plates. The surface pH was measured by means of a pH paper placed on the surface of the swollen patch. A mean of three readings was recorded.

*Folding Endurance Test.* This test was done by repeatedly folding the patch at the same place up to maximum 300 times or till it broke [17].

*Swelling Index. *After determination of original film weight and diameter, the samples were allowed to swell on the surface of agar plate kept in an incubator maintained at 37°C. Increase in the weight or diameter was calculated after the preset time interval [18]. The measurement of diameter of patch was done by using microscope after 1 h interval for 5 h. The percentage swelling, %*S*, was calculated using the following equation:

(3) $$\begin{array}{c} \%S\;=\;\left(\frac{{W_{t}-W}_{0}}{W_{0}}\right)*100. \end{array}$$

*W*_*t*_ is weight of the patch after time *t* and *W*_0_ is the initial weight at zero time. 

*In Vitro Bioadhesive Test.  In vitro *bioadhesive test of the prepared films was examined using chicken pouch as a model mucosal membrane [19]. The tissue was obtained from chicken after slaughter, removed from its contents and surface fats, and stored frozen in simulated artificial saliva solution. It has thawed to room temperature before study. A rectangular piece of the tissue was cut and glued with adhesive on the ground surface of the two tissue holders made of Plexiglas. One centimetre of the buccal film was placed between the two tissue surfaces and put into contact with each other with uniform and constant light pressure between fingers for one minute to facilitate adhesive bonding. The upper tissue holder was allowed to hang on an iron stand with the help of an aluminium wire fastened with a hook provided on the back of the holder. A preweighed light weight polyethylene bag was attached to the hook on the back of the lower tissue holder with aluminium wire. After preload time of one minute, water was added to the polyethylene bag through an intravenous infusion set at a rate of 2.0 drops per second until the lower tissue detached due to the heavy weight of the water infused. The water collected in the bag was measured and expressed as weight (gram force) required for the detachment, using the following equation:

(4) $$\begin{array}{c} \text{Detachment}\;\text{stress}\left(\frac{\text{dyne}}{\text{c}{\text{m}}^{2}}\right)=\left(m*g/A\right), \end{array}$$

where *m* is the weight of the water infused at detachment, *g* the acceleration due to gravity considered as 980 cm/s^2^, and *A* the area of tissue exposed (cm^2^).

*Determination of In Vitro Residence Time*. The *in vitro *residence time was determined using a locally modified USP disintegration apparatus, based on the apparatus applied by Nakamura et al. [20]. The disintegration medium was composed of 800 mL pH 6.6 isotonic phosphate buffer (IPB) maintained at 37 ± 0.5°C. A porcine buccal mucosa, 3 cm length, was glued to the surface of a glass slab, vertically attached to the apparatus. The mucoadhesive patch was hydrated from one surface using 15 *μ*L pH 6.6 IPB and then the hydrated surface was brought into contact with the mucosal membrane. The glass slab was vertically fixed to the apparatus and allowed to move up and down so that the patch was completely immersed in the buffer solution at the lowest point and was out at the highest point. The time necessary for complete erosion or detachment of the patch of each batch from the mucosal surface was recorded. 

*Vapour Transmission Test (VTR).* Vapour transmission method was employed for the determination of vapour transmission from the patch [20]. Glass-bottle (length = 5 cm, narrow mouth with internal diameter = 0.8 cm) filled with 2 g anhydrous calcium chloride and an adhesive (Feviquick) spread across its rim was used in the study. The patch was fixed over the adhesive and the assembly was placed in a constant humidity chamber, prepared using saturated solution of ammonium chloride and maintained at 37 ± 2°C. The difference in weight after 24 h, three days, and 1 week was calculated. The experiments were carried out in triplicate and vapour transmission rate was obtained as follow: VTR = (Amount of moisture transmitted)/(area × time). 

*Content Uniformity. *The medicated patch was allowed to dissolve in 100 mL phosphate buffer, pH 6.8. The amount of drug in the solution was measured spectrophotometrically at *λ*_max_ of 285 nm. 

*In Vitro Drug Release Study.* The release study was done in the Keshery-Chien diffusion cell using pH 6.8 buffer medium [21]. The cellophane membrane was carefully mounted in between the two compartments of a Keshery-Chien diffusion cell with internal diameter of 2.1 cm (3.46 cm^2^ area) with a receptor compartment volume of 12 mL of solution containing phosphate buffer pH (6.8) were placed in the receptor compartment. Temperature was maintained at 37 ± 2°C. Firstly the release study was done for the patches of different polymer concentrations of both HPMC K100CR and HPMC K15 and the result is shown in Figures 6 and 7. The patches Hb2 and Pb4 were selected for final formulation due to their release profile. Release study using egg membrane also conducted for the optimised formulations. The withdrawals were compensated using equal volumes of phosphate buffer pH 6.8 kept at the same temperature. The concentration of drug released in the medium was assayed spectrophotometrically at 285 nm after suitable dilution with the diffusion medium phosphate buffer pH 6.8 whenever necessary. The experiment was carried out continuous up to 10 h. All the experiments were conducted in 3 replicates. 

#### 2.4.4. *Ex Vivo* Release Study

*Tissue Preparation. *The goat buccal mucosal tissue was obtained immediately post sacrifice from a local slaughter house (Kothrude, Pune, India) and transported to the laboratory in isotonic phosphate buffer, pH 7.4. The buccal mucosal tissue was rinsed with isotonic phosphate buffer. The mucosa was removed from the underlying muscular layer by cutting the loose connective fibres with a scalpel. Circular pieces were then punched out. The excised mucosa was immersed in isotonic saline at 60°C for 1 min and the epithelium was then peeled away from connective tissue. Samples were briefly dipped in deionised water, dried on a cellulose filter and frozen at −20°C until use (no more than 3 weeks). The *ex vivo *release study was done same as *in vitro* release study but in *ex vivo *study in case of cellophane membrane goat buccal mucosal membrane was used. The optimized patches were used for exvivo study.

*Stability Studies and Aging.* Plain and drug loaded patches were packaged in aluminium foil and stored in glass bottles closed with screw caps. These bottles were subjected to accelerated stability testing using stability chambers (Newtronic, India) maintained at 25°C/60% RH, 30°C/65% RH, & 40°C/75% RH for 3 months and evaluated for their physical changes such as colour and texture, drug-polymer interaction, drug, content and diffusion study at 1st, 2nd and 3rd month.

## 3. Results and Discussion

### 3.1. Drug Characterization

The melting point of darifenacin was found to be 229–2360°C. Darifenacin is freely soluble in chloroform (13.4 mg/mL). It is slightly soluble in methanol (8.8 mg/mL) and ethanol (8 mg/mL) and is practically insoluble in water. IR study of darifenacin show peaks at 3463 cm^−1^ (N–H stretching), 1663 cm^−1^ (C=O ester stretch), 1584 cm^−1^ (–C=C aromatic). UV analysis of darifenacin using different solvents show absorption maxima at 285 nm and calibration curves were prepared on the absorbance at 285 nm.

### 3.2. Darifenacin-Hp*β*CD Complexation

**Figure 1:**
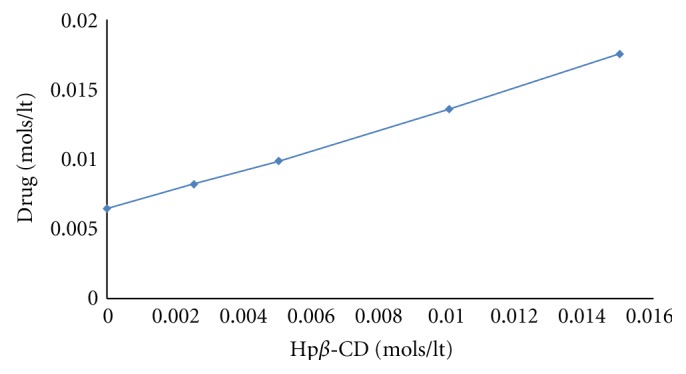
Phase solubility diagram of the darifenacin-Hp*β*-CD system.

The phase solubility diagram obtained for Darifenacin-Hp*β*CD is represented in Figure 1. The shape of the solubility diagram followed an *A*_*L*_ type system. The apparent stability constant, *K*_*s*_, was calculated to be 465.301 M^−1^. This result is in good agreement with the earlier reported value. 

### 3.3. Gibbs Free Energy Change (Δ*G*_tr_°)

The negative nature of the Gibbs free energy (Table 3) changes (Δ*G*_tr_°; 0.614, −1.08, −1.867, −2.506) 0.0025, 0.05, 0.01,0.015 moles/L of water respectively are indicative of the spontaneity of the process. The endothermic heats of solution further explain the increase in solubility with temperature. 

### 3.4. Stability Study of Drug in Different Solvents and Calibration Curve

Darifenacin shows neither any shift of absorption maxima nor any decrease of absorbance in the solvents at a particular concentration which indicates that drug is stable in distilled water, pH 6.8 buffer, and 0.1 N HCl. Regression equation (*y* = *mx* + *c*) for the drug in different solvents at 285 nm was as follows. Slope = 0.005, intercept = 0.002667, *R* ^2^ = 0.9997 in distilled water, slope = 0.00467, intercept = 0.0027, *R* ^2^ = 0.999 in 0.1 N HCl and slope = 0.004, intercept = 0.0028, *R* ^2^ = 0.998 in pH 6.8 buffer.

### 3.5. Percentage Yield

Percentage yield (Table 4) is calculated and found to be 42–87% least in spray drying and maximum in physical mixture complexes. The yield is much lower in spray drying which may be due to the improper operating or due to less efficiency of the instrument.

### 3.6. Drug Content of Complex

Drug content of complexes (Table 4) was found to be within 86−95%. 

### 3.7. Saturation Solubility Studies

Saturation solubility (Table 4) of pure drug was 3.14 mg/mL and that of complexes found to be 8.24–20.57 mg/mL and percentage increase in solubility of the drug in co-evaporation complex was 386% and maximum was 659% for freeze dried complex. Though saturation solubility and percentage increase in solubility of freeze dried and spray dried complexes are more but co-evaporation complex was chosen for formulation as freeze drying and spray drying process are expensive and sophisticated and may also increase the formulation costs.

### 3.8. Differential Scanning Calorimetry (DSC)

**Figure 2:**
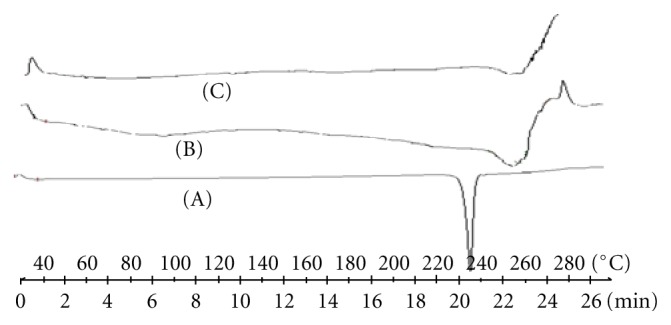
Differential scanning calorimetry (DSC) of darifenacin and Hp*β*CD complex. (a) Darifenacin, (b) physical mixture, (c) coevaporation complex.

DSC thermograms are shown in Figure 2. The DSC curve of darifenacin showed an endothermic event as a melting peak with the onset temperature of 235.02^*◦*^C, indicating a crystal polymorph form. The appearance of a peak, corresponding to darifenacin melting, was also evident in the thermogram of the physical mixture. The appearance of endothermic peak with decreased intensity in the thermogram of the co-grounding complex could be attributed to the inclusion of darifenacin in the Hp*β*-CD cavity. On the other hand, the absence of DSC signal indicates amorphous character of the co-evaporation complex.

### 3.9. X-Ray Diffractometry

**Figure 3:**
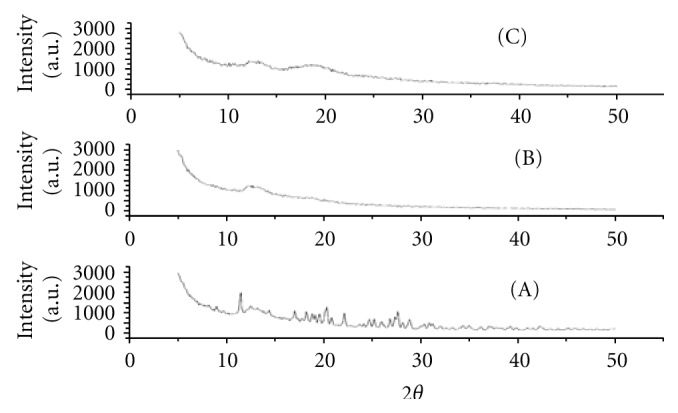
X-ray diffractometry of (a) darifenacin, (b) coevaporation complex and (c) physical mixture complex.

The X-ray diffractometry studies (Figure 3) revealed the crystalline nature of darifenacin. Darifenacin shows major peaks at 2*θ* values 11.3, 11.4, 11.5, 17.00, 18.2, 20.1, 20.2, 20.3, 25.2, 26.8, 27.3, 27.6, 30.3, and 30.8. The peaks observed were less intense than those of the drug in physical mixture and co-evaporation complex. The diffractogram of the co-evaporation complex showed no darifenacin crystal signals, demonstrating the amorphous nature of the product. The study of these spectra indicated that degree of crystallinity was decreased by complexation with Hp*β*-CD.

### 3.10. FT-IR Spectra

**Figure 4:**
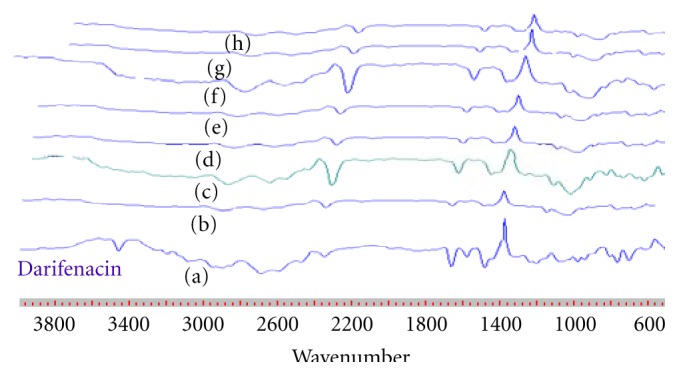
FTIR spectra of darifenacin and complexes. (a) Darifenacin, (b) physical mixture, (c) co-grounding complex, (d) coevaporation, (e) freeze dried, (f) spray dried, (g) melting method, (h) kneading method.

FT-IR spectra are presented in Figure 4. Darifenacin spectra showed the band at 3463 cm^−1^. From Figure 4 it was found that inclusion complexes of darifenacin showed spectra with broader bands at 1663 cm^−1^ and 3463 cm^−1^, suggesting the formation of hydrogen bonds between the carbonyl group and N–H group of darifenacin with the hydroxyl group of the host cavity. 

### 3.11. Dissolution Study of Complexes

**Figure 5:**
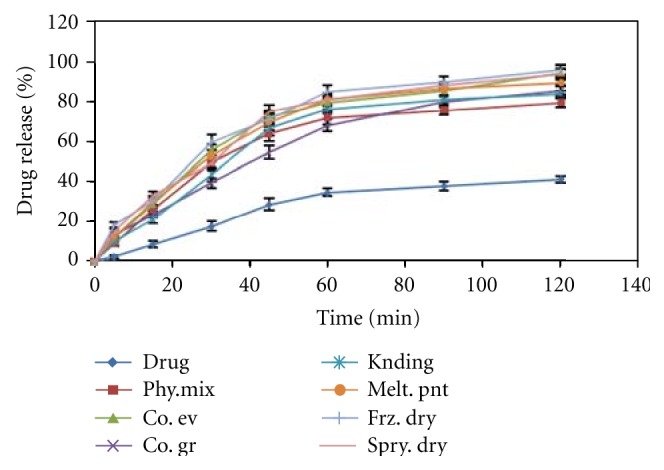
Dissolution profile of different complexes of darifenacin-Hp*β*-CD in pH 6.8 buffer.

Dissolution study of the complexes (Figure 5) shows that co-evaporation; freeze dried and spray dried complexes show drug release above 94% in 2 h. In other complexes release is less than 88% and in pure drug release is below 43% in 2 hours. So among the complexes showing higher dissolution co-evaporation complex is selected for formulation as freeze drying and spray drying is expensive and sophisticated processes.

### 3.12. Evaluation of Buccoadhesive Patches

Buccoadhesive patches of darifenacin alone or in the complex form were prepared using HPMC K100CR and of HPMC K15. All the formulations contained the co-evaporation complex. (Table 1).

#### 3.12.1. Thickness Uniformity

All the patches have uniform thickness (Tables 5 and 6) throughout. Average thickness found was about 0.81 mm.

#### 3.12.2. Surface pH

The surface pH of all formulations (Tables 5 and 6) was within 5.5–6.8 units of the neutral pH and hence no mucosal irritation was expected and ultimately achieves patient compliance.

#### 3.12.3. Folding Endurance

Films did not show any cracks even after folding for more than 300 times. Hence it was taken as the end point. Folding endurance did not vary when the comparison was made between plain films and drug loaded films.

#### 3.12.4. Weight Uniformity

 The patches were found (Tables 5 and 6) to be uniform. The average weight of the patch was found to be about 65.74 mg.

#### 3.12.5. Content Uniformity

The results of content uniformity indicated that the drug was uniformly dispersed.

#### 3.12.6. Swelling Studies

The swelling of the patches was observed in phosphate buffer solution (pH 7.4) and shown in Tables 5 and 6. The patches show swelling index 38%–49% in HPMC K100CR patches and for HPMC K15 patches it was 25.4%–37.22%. The results indicate that swelling index increases with increase in polymer concentration. 

### 3.13. *In Vitro *Bioadhesive Test

In general, muccoadhesion is considered to occur in three major stages: wetting, interpenetration, and mechanical interlocking between mucus and polymer. The strength of mucoadhesion is affected by various factors such as molecular mass of polymers, contact time with mucus, swelling rate of the polymer, and the biological membrane used in the study. HPMC K100 M CR patches show (Table 7) adhesion forces between 0.099 and 0.146 N. HPMC K15 patches (Table 8) have force of adhesion between 0.094 and 0.122 N. The study indicates with increase in polymer concentration the mucoadhesive strength increases. The study also indicates that HPMC K100CR patches have more adhesive force than HPMC K15 patches. So it implies adhesive force depends on the viscosity of the polymers.

### 3.14. *In Vitro* Residence Time

Observations related to the *in vitro *residence time including detachment as well as erosion for patches both plain and medicated, indicated adequate attachment to the mucosal surface without erosion that is, 5.8 to 6.6 hours. HPMC mucoadhesion time always resulted high, because the polymer has higher swelling capacity and tends to retain its structure better at high molecular weight of polymer.

### 3.15. Vapour Transmission Rate (Table 9)

Formulation Hb2 and Pb4 shows  1.63 × 10^−3^ ± 0.56 × 10^−3^ g cm^−2 ^ h^−1^ and 1.53 × 10^−3^ ± 0.55 × 10^−3^ g cm^−2 ^ h^−1^ show highest permeation respectively, on day seven while less permeation, was found in Hb3 and Pb3 formulations, that is, 0.83 × 10^−3^ ± 0.07 × 10^−3^ g cm^−2^  h^−1^ and 0.78 × 10^−3^ ± 0.22 × 10^−3^ respectively, indicating the presence of higher concentration of water insoluble HPMC. 

### 3.16. Content Uniformity

The results of content uniformity indicated that the drug was uniformly dispersed. Recovery was possible to the tune of 89 to 95%.

### 3.17. *In Vitro* Release

**Figure 6:**
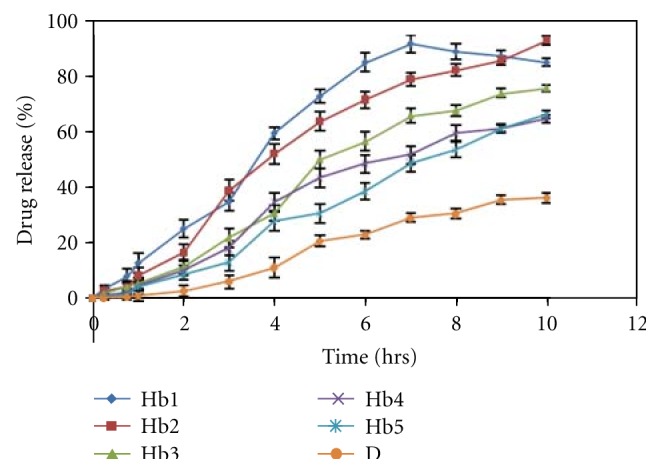
*In vitro* release profile of different formulations of HPMC K100 M CR containing Hp*β*-CD-darifenacin complex. Hb1–Hb5 formulations of HPMC K100 M CR containaing 1%–5% of the polymer, D-formulation containing pure drug.

**Figure 7:**
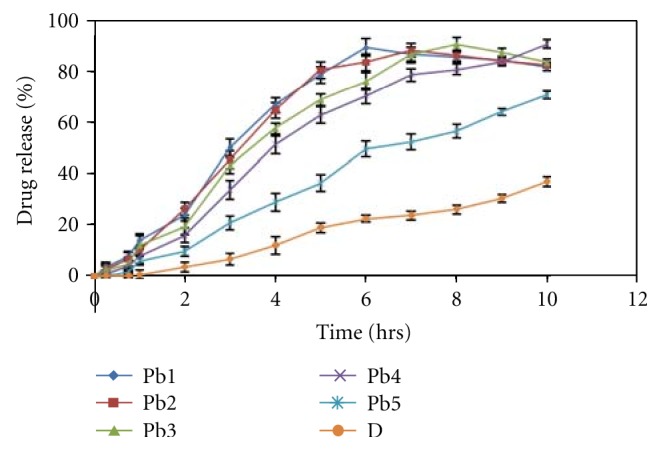
*In vitro* release profile of different formulations of HPMC K15 containing Hp*β*-CD-darifenacin complex. Pa1–Pa5 formulations of HPMC K15 containaing 1%–5% of the polymer, D-formulation containing pure drug.

The release data of darifenacin from all the patches are given in Figures 6 and 7. A perusal to Figure 6 indicated that the drug release from Hb2 (2%) patch was above 90% in 10 hours. Hb1 had also given 90% release but that was within 6 and 7 h. Formulations Hb3, Hb4, and Hb5 show below 74% release in 10 h. So to get the desired drug release, Ha2 formulation was finalised for egg membrane and *ex vivo *studies. The diffusion study of the different patches of HPMC K15 shown in Figure 7 indicates that formulation Pb4 (4%) shows about 90% drug release in 10 hours. Formulation Pb1, Pb2, Pb3 and show above 90% drug release but they were within 6-7 hours which was not the desired result. Formulation Pb5 shows less than 71% release with in 10 hours. So Pa4 formulation is finalised for further *ex vivo* studies. The diffuson release parameters for HPMC K100M CR and HPMC K15 formulations of buccal patch is as show in Tables 10 and 11.

### 3.18. *Ex Vivo* Drug Release

**Figure 8:**
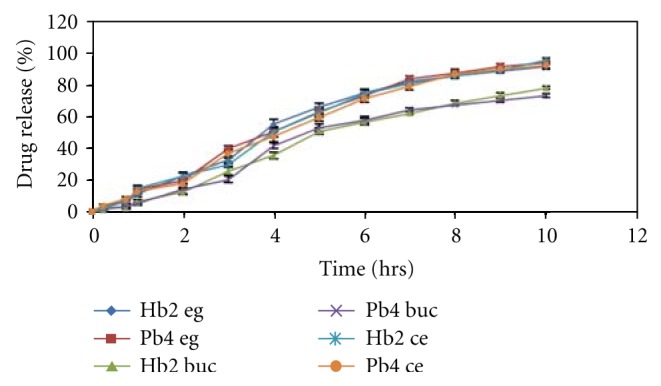
Drug diffusion study through goat buccal membrane and egg membrane (eg: egg membrane diffusion, buc: buccal membrane diffusion, ce: cellophane membrane).

*Ex vivo *release study performed by goat buccal membrane shows 78% to 73% in 10 h from the optimized patches of HPMC K100 M CR and HPMC K15 shown in Figure 8. Drug diffusion rate through egg membrane was also studied in Figure 8 which shows similar diffusion (above 90% release in 10 hours) as through cellophane membranes.

### 3.19. Stability Studies and Aging

**Figure 9:**
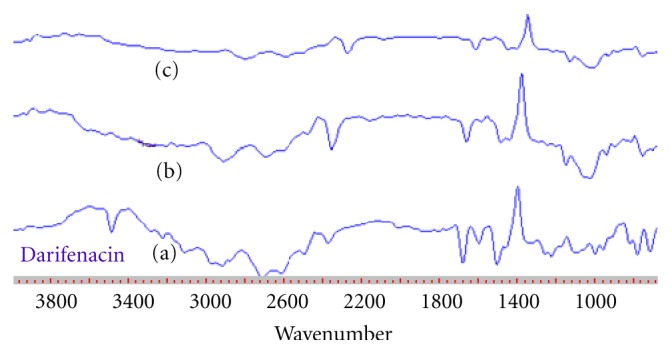
FTIR spectra of darifenacin and formulations. (a) Darifenacin, (b) drug-Hp*β*-CD complex with HPMC K15, (c) drug-Hp*β*-CD complex with HPMC K100 M CR.

The data analysis during and after stability studies up to 3 months indicates that the drug molecules remain unchanged in the patches and shows no interaction with the polymers (Figure 9) and no degradation. Percentage drug content in 1st, 2nd, and 3rd month analysis was within 94–97%. Diffusion study of the optimised formulations in the subsequent 3 months was same as the initial *in vitro *and *ex vivo *release studies. 

## 4. Conclusion 

 Water-soluble carrier beta cyclodextrin was complexed with darifenacin by different techniques namely co-evaporation, co-grinding, kneading, closed melting method, and spray drying and freeze drying methods. Among the above methods co-evaporation method was used for buccal patch formulation.

Phase-solubility studies revealed *A*_*L*_ type of curves for each carrier, indicating linear increase in the drug solubility with carrier concentration. All the complexes showed dissolution improvement in comparison to pure drug to varying degrees, where Hp*β*-CD was used as the most promising carrier. Solid-state characterization studies revealed that the drug crystallinity played pivotal role in governing the solubility characteristics of the drug. As with the Hp*β*-CD-darifenacin complex sample, XRD analysis of the product confirmed a characteristic amorphous pattern, FTIR spectra did not show any evidence for a chemical interaction between the components, and DSC showed the absence of melt endotherms, ostensibly accounting for enhancement in dissolution rate. Therefore, it was concluded that co-evaporation method of  darifenacin with Hp*β*-CD resulted in amorphous products. The stability studies indicate that Hp*β*-CD systems were stable for period of study (3 months).

 Hb2 formulation of HPMC K100 M CR and Pb4 formulation of HPMC K15 show above 90% release in 10 h. Muccoadhesive strength of these patches was sufficient and also show required *in vitro *residence time. Stability study of the drug in these formulations did not show any chemical interaction. *In vitro* drug release and *ex vivo* release were also in agreement with the prestability release data. 

## Figures and Tables

**Table 1:** HPMC K100CR formulations.

Serial no.	Formulation batch	Darifenacin-Hp*β*-CD complex (mg)	HPMC K100M CR (%)	Propylene glycol (%)
1	Hb 1	32	1	20
2	Hb 2	32	2	20
3	Hb 3	32	3	25
4	Hb 4	32	4	30
5	Hb 5	32	5	30

**Table 2:** HPMC K15 formulations.

Serial no.	Formulation batch	Darifenacin-Hp*β*-CD complex (mg)	HPMC K15 (%)	Propylene glycol (%)
1	Pb 1	32	1	20
2	Pb 2	32	2	20
3	Pb 3	32	3	25
4	Pb 4	32	4	25
5	Pa 5	32	5	30

**Table 3:** Gibbs free energy of transfer (Δ*G*_tr_°) for solubilization process of darifenacin in aqueous solutions of Hp*β*-CD at 37°C.

Moles of *β*-CD in water	Δ*G*_tr_° (kJ/mol)
0.0025	−0.614
0.005	−1.08
0.01	−1.867
0.015	−2.506

**Table 4:** Evaluation parameter of complexes.

Drug: Hp*β*-CD Complex	% Practical Yield	% Drug Content	Saturation Solubility (mg/mL)	% Increase in solubility
Drug	—	—	3.14	—
Physical mixture	87.17 ± 2.17	92.11 ± 1.34	8.58	273.24 ± 4.64
Kneading	84.12 ± 1.83	95.32 ± 2.14	8.24	262.42 ± 5.79
Co-grounding	85.03 ± 1.98	89.59 ± 1.58	8.31	264.64 ± 3.98
Co-evaporation	78.17 ± 1.71	93.88 ± 1.74	12.13	386.30 ± 4.55
Melting method	82.43 ± 2.76	88.7 ± 1.89	9.17	292.03 ± 5.75
Freeze-Drying	86.12 ± 2.49	95.24 ± 3.57	20.71	659.55 ± 8.47
Spray Drying	42.54 ± 3.27	86.88 ± 2.63	16.54	526.75 ± 7.33

**Table 5:** Evaluation of HPMC K100CR patches.

Formulation code	Thickness (mm)	Weight uniformity (mg)	Surface pH	Content uniformity (%)	*In vitro* drug release (%) (10 hrs)	Swelling index (% weight increase after 3 hrs)
Hb1	0.69 ± 0.05	58.69 ± 0.87	6.13 ± 0.059	91.86 ± 1.57	84.51 ± 1.863	38.45 ± 0.43
Hb2	0.85 ± 0.07	68.63 ± 0.676	6.66 ± 0.085	95.93 ± 2.3	92.487 ± 2.04	39.57 ± 1.78
Hb3	0.82 ± 0.12	71.56 ± 0.536	6.21 ± 0.07	93.28 ± 1.45	75.42 ± 1.487	44.01 ± 1.88
Hb4	0.87 ± 0.04	75.41 ± 0.674	5.54 ± 0.075	100.22 ± 3.16	64.31 ± 2.32	46.75 ± 2.73
Hb5	1.08 ± 0.21	79.82 ± 0.615	5.92 ± 0.065	90.38 ± 1.22	66.02 ± 2.24	49.48 ± 3.04

**Table 6:** Evaluation of HPMC K15 patches.

Formulation code	Thickness (mm)	Weight uniformity (mg)	Surface pH	Content uniformity (%)	*In vitro *drug release (%) (10 hrs)	Swelling index (% weight increase after 3 hrs)
Pb1	0.67 ± 0.07	60.35 ± 0.46	5.93 ± 0.07	94.18 ± 1.81	82.45 ± 1.68	25.4 ± 0.61
Pb2	0.71 ± 0.05	65.33 ± 0.97	5.97 ± 0.056	96.87 ± 2.13	83.26 ± 2.01	27.13 ± 1.03
Pb3	0.74 ± 0.08	67.11 ± 0.73	6.78 ± 0.055	101.37 ± 3.14	84.36 ± 2.44	30.23 ± 1.31
Pb4	0.79 ± 0.07	71.02 ± 1.24	5.99 ± 0.047	93.67 ± 2.14	91.54 ± 2.15	33.42 ± 1.18
Pb5	0.82 ± 0.05	73.57 ± 1.39	6.08 ± 0.074	97.22 ± 1.87	71.44 ± 2.37	37.22 ± 2.54

**Table 7:** Bioadhesive parameters of HPMC K100M CR patches.

Formulation code	Bioadhesive strength (g)	Force of adhesion (N)	Bond strength (N m^−2^)
Hb1	10.15 ± 0.67	0.099	994.7
Hb2	12.02 ± 1.38	0.117	1177.96
Hb3	12.62 ± 1.22	0.123	1236.76
Hb4	13.87 ± 1.42	0.135	1359.26
Hb5	14.98 ± 1.54	0.146	1468.804

**Table 8:** Bioadhesive parameters of HPMC K15 patches.

Formulation code	Bioadhesive strength (g)	Force of adhesion (N)	Bond strength (N m^−2^)
Pb1	9.65 ± 0.78	0.0945	9457.13
Pb2	10.38 ± 0.64	0.101	1017.24
Pb3	10.86 ± 0.93	0.106	1064.28
Pb4	11.73 ± 1.25	0.114	1149.54
Pb5	12.44 ± 1.43	0.122	1228.92

**Table 9:** Vapour transmission rate of prepared mucoadhesive buccal patches.

Formulation code	Moisture vapour transmission (g·cm^−2^·h^−1^)		
	Day 1	Day 3	Day 7
Hb1	5.32 × 10^−3^ ± 0.23 × 10^−3^	2.24 × 10^−3^ ± 0.26 × 10^−3^	0.93 × 10^−3^ ± 0.39 × 10^−3^
Hb2	7.54 × 10^−3^ ± 2.32 × 10^−3^	2.14 × 10^−3^ ± 0.81 × 10^−3^	1.63 × 10^−3^ ± 0.56 × 10^−3^
Hb3	7.44 × 10^−3^ ± 0.44 × 10^−3^	2.70 × 10^−3^ ± 0.53 × 10^−3^	0.83 × 10^−3^ ± 0.07 × 10^−3^
Hb4	8.81 × 10^−3^ ± 1.82 × 10^−3^	3.87 × 10^−3^ ± 0.72 × 10^−3^	1.22 × 10^−3^ ± 0.72 × 10^−3^
Hb5	9.42 × 10^−3^ ± 2.32 × 10^−3^	3.77 × 10^−3^ ± 0.87 × 10^−3^	1.33 × 10^−3^ ± 0.46 × 10^−3^
Pb1	5.76 × 10^−3^ ± 0.62 × 10^−3^	2.18 × 10^−3^ ± 0.17 × 10^−3^	1.33 × 10^−3^ ± 0.08 × 10^−3^
Pb2	6.88 × 10^−3^ ± 1.45 × 10^−3^	2.65 × 10^−3^ ± 0.81 × 10^−3^	1.50 × 10^−3^ ± 0.36 × 10^−3^
Pb3	7.32 × 10^−3^ ± 1.23 × 10^−3^	2.68 × 10^−3^ ± 0.47 × 10^−3^	0.78 × 10^−3^ ± 0.22 × 10^−3^
Pb4	8.47 × 10^−3^ ± 0.80 × 10^−3^	3.89 × 10^−3^ ± 0.46 × 10^−3^	1.53 × 10^−3^ ± 0.55 × 10^−3^
Pb5	8.35 × 10^−3^ ± 0.93 × 10^−3^	3.91 × 10^−3^ ± 0.98 × 10^−3^	0.90 × 10^−3^ ± 0.50 × 10^−3^

**Table 10:** Diffusion parameter of HPMC K100M CR formulations.

Complexes	Best fit model	*n*	*R* ^2^
D	Zero order	1.0032	0.9947
Ha1	Peppas	0.86378	0.9378
Ha2	Peppas	0.9642	0.9858
Ha3	Peppas	0.9326	0.9943
Ha4	Zero order	1.021	0.9842
Ha5	Zero order	1.001	0.9955

**Table 11:** Diffusion parameter of HPMC K15 formulations.

Complexes	Best fit model	*n*	*R* ^2^
D	Zero order	1.002	0.9941
Pa1	Peppas	0.8673	0.9854
Pa2	Peppas	0.8534	0.9887
Pa3	Peppas	0.9236	0.9971
Pa4	peppas	0.8794	0.9957
Pa5	Zero order	1.03	0.9934
